# Effects of Skeletally Supported Anterior en Masse Retraction with Varied Lever Arm Lengths and Locations in Lingual Orthodontic Treatment: A 3D Finite Element Study

**DOI:** 10.1155/2021/9975428

**Published:** 2021-05-17

**Authors:** Mohammad Ghannam, Beste Kamiloğlu

**Affiliations:** Department of Orthodontics, Near East University, Nicosia, Northern Cyprus, Mersin 10, Turkey

## Abstract

**Objective:**

This study is aimed at analyzing different points of force application during miniscrew supported en masse retraction of the anterior maxillary teeth to identify the best line of action of force in lingual orthodontic treatment.

**Materials and Methods:**

Three-dimensional (3D) finite element models were created to stimulate en masse retraction with different heights and positions of the miniscrew and lever arm to change the force application points; a 150 g retraction force was applied from the miniscrew to the lever arms, and the initial tooth displacements were analyzed.

**Results:**

Lingual crown tipping and occlusal crown extrusion were seen at all heights and positions of the miniscrew and lever arm, but when the miniscrew height was at 8 mm and the power arm was located between the lateral incisors and canines, these tipping patterns were less than those obtained with a 4.5 mm high miniscrew and a lever arm located distal to the canines.

**Conclusion:**

All miniscrew heights and lever arm positions showed initial lingual crown tipping and labial root tipping with occlusal crown extrusion. However, the 8 mm miniscrew height and the lever arm located between the lateral incisor and canine showed fewer amounts of these tipping patterns than a 4.5 mm miniscrew height and lever arm located distal to the canines. Therefore, this could be the preferred point of force application during en masse retraction in lingual treatment with additional torque control methods.

## 1. Introduction

With the increasing adoption of orthodontic treatment among adult patients, especially female, patient esthetic demands have been reportedly increasing [1]. Lingual appliances address the esthetic requirements for such patients by attaching to the lingual surface of the teeth [2]. The first generation of lingual orthodontic appliances was introduced by Kurz in the 1970s with his lingual edgewise appliance with an anterior bite plan and mesh pads to adapt to the tooth lingual surface and pretorqued archwire [3]. Subsequently, Fujita introduced lingual mushroom-shaped wires that were developed to overcome the difference in labiolingual thickness between the anterior and posterior teeth [4].

3D models extracted from cone beam computed tomography (CBCT) scans of craniofacial structures including maxillary and mandibular dentitions made it possible to evaluate the treatment outcomes in all three planes of space [5]. Leonardi et al. and Perillo et al. used 3D models derived from CBCT scans to evaluate skeletal changes or investigate craniofacial characteristics among different ethnic groups which show the accuracy and reliability of 3D techniques [5, 6].

To obtain successful outcomes in any orthodontic treatment, a good knowledge of biomechanics is mandatory, since the treatment's outcome primarily depends on the biomechanical principles applied during the treatment [7]. En masse retraction of the six maxillary anterior teeth as one unit to close an extraction space is a standard clinical practice procedure. To control the anchorage in the posterior teeth, miniscrews can be used to achieve maximal retraction of the anterior teeth during lingual orthodontic treatment [8]. Variations in the direction of the applied retraction will result in multiple movement patterns of the anterior teeth, which may lead to unfavorable outcomes. Thus, it is crucial to understand the resultant effects of the different movement patterns during en masse retraction of the anterior maxillary teeth with the use of miniscrews in lingual orthodontics [8].

A previous study [8] investigated the movement tendencies of the maxillary anterior teeth during lingual en masse retraction with a single miniscrew height and two varied lever arm locations using the finite element method. Applying retraction force from different miniscrew heights could generate new movement patterns which could be favorable for function and esthetics. Three-dimensional finite element analysis can be used as an efficient computer simulation technique to imitate the orthodontic force application and analyze the resultant biomechanical actions of the teeth [9].

In light of this rationale, the miniscrew heights and lever arm locations and positions were varied to widen the possibility of determining favorable points of force application. A 3D finite element model with the alveolar bone, periodontal ligament, teeth, and lingual orthodontic system was constructed in the current study. The aim was to analyze the resultant displacement patterns after applying retraction force to identify the best line of action of force for functionally and esthetically pleased outcomes.

## 2. Material and Methods

Three-dimensional geometric models with finite element analysis that contained the first premolar extraction space and lingual system were used. To establish the 3D finite element models with the maxilla and the maxillary dentition, computed tomography scans of an adult male's dry skull were acquired from the Visible Human Project® (US National Library of Medicine, Bethesda, MD). The scans were then modified into a meshwork using the VRMesh Design (Virtual Grid Inc., Bellevue City, WA) software. The periodontal ligament structure was formed evenly with 0.25 mm thickness [10]. Around the PDL and on all outer bone surfaces, the cortical bone was formed evenly with 1 mm thickness, and the cancellous bone filled the remaining bone areas [11].

Next, 0.018 × 0.025-inch slot ORMCO 7th generation lingual brackets, 0.016 × 0.022-inch stainless steel ORMCO (Ormco, Glendora, CA), preformed mushroom-shaped lingual archwires, and power arms and miniscrews (Ormco VectorTAS orthodontic implant; length of the miniscrew, 10.7 mm) were modeled with the Rhinoceros 4.0 (McNeel & Associates, Seattle, WA) software using the original shapes and dimensions. The lever arm varied between two locations with three different heights from the archwire plane: 6 mm, 8 mm, and 10 mm. The first location was mesial to the canine in the midpoint between the lateral incisor and canine. The second location was distal to the canine. The lever arms were extended toward the gingiva and palatal mucosa. The miniscrews were located between the first and second maxillary molars at two different heights from the archwire plane, 4.5 mm and 8 mm [12]. The maxillary first premolars were removed to accommodate the en masse retraction.

**Figure 1:**
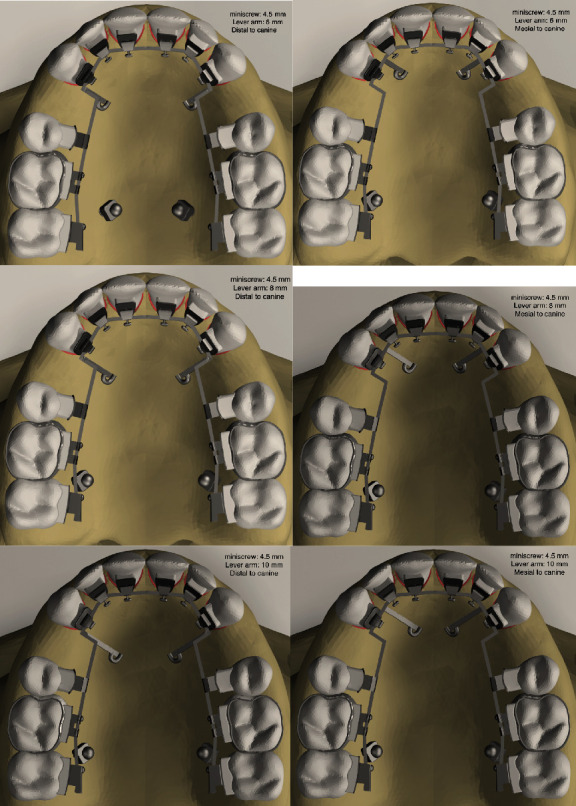
The generated models with miniscrew height 4.5 mm and varied lever arm.

**Figure 2:**
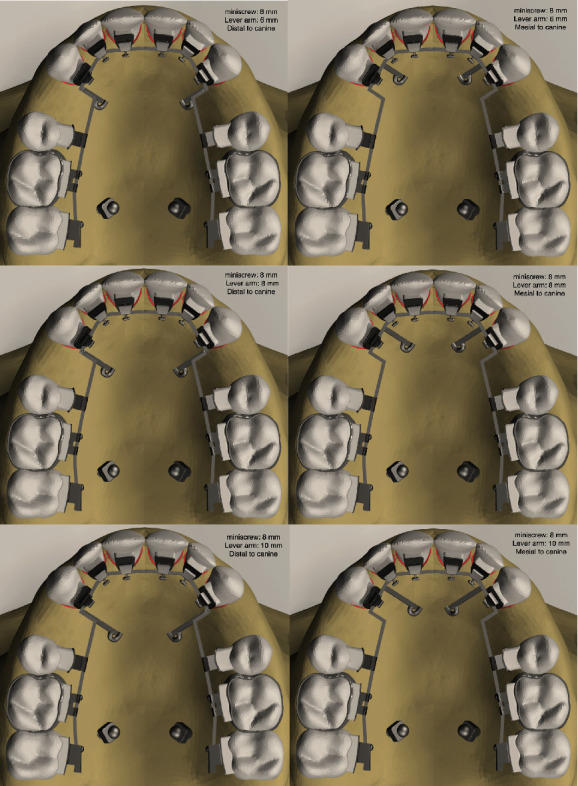
The generated models with miniscrew height 8 mm and varied lever arm.

Twelve models were formed according to varied locations and heights of lever arms and miniscrews and transferred into ALGOR FEMPRO software (ALGOR, Inc., Pittsburgh, PA) to generate the finite element analysis (Figures 1 and 2). In all models, the structure of teeth, periodontal ligament, cortical and cancellous bone, brackets, and archwire was formed using 8-node brick elements. In areas close to the center of the structures in the models, 6-node wedge, 5-node pyramid, and 4-node tetrahedral elements were also used. The node and element numbers are illustrated in Table 1. All structures in the models were determined as linear elastic, homogeneous, and isotropic [13].  Table 2 shows the material properties that were used [14].

The boundary conditions were determined in the areas where the maxillary bone ends.

**Figure 3:**
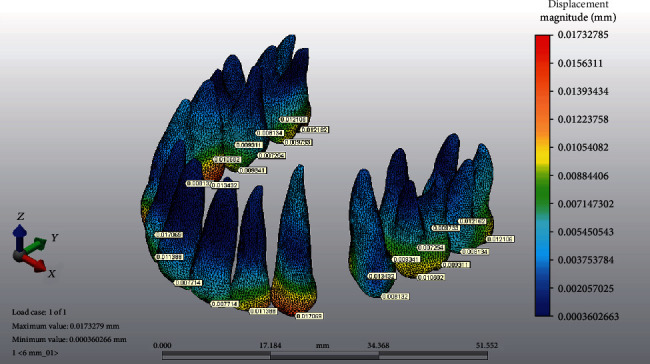
Example of measurements of the reference points on the incisal edges of the crowns.

**Figure 4:**
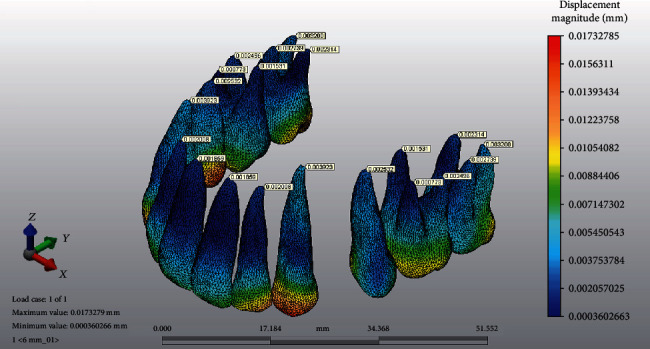
Example of measurements of the reference points on the root apices.

The force application points were at a vertical distance of 6, 8, and 10 mm from the archwire plane on the lever arm, and a 150 g retraction force was applied from the miniscrew to the identified point on the lever arm. In order to analyze the initial displacement patterns, a local 3D coordinate system was used. The coordinate system incorporated the *X*, *Y*, and *Z* axes perpendicular to each other. The *X*-axis indicated labiolingual displacements: +lingual, -labial; the *Y*-axis indicated the mesiodistal direction: +mesial, -distal; and the *Z*-axis indicated the vertical direction: +occlusal, -apical. Reference points were placed on the incisal edges of crowns (Figure 3) and root apices (Figure 4) of the sixth anterior teeth in order to measure tooth displacement. The resultant initial displacement of these nodes on the *X*, *Y*, and *Z* axes after force application was analyzed.

## 3. Results

In measurements performed after application of the retraction force with the lever arm located distal to the canine with the miniscrew height at 4.5 mm, when the length of the lever arm was 6 mm, the maxillary incisors and canines showed lingual crown tipping, and labial root tipping increased significantly from the central incisors to the canines. The maxillary incisors and canines also showed mesial crown tipping, and distal root tipping increased significantly from the central incisors to the canines. The maxillary incisors and canines showed both crown and root occlusal extrusion (Table 3). At a lever arm length of 8 mm, the maxillary incisors and canines still showed lingual crown tipping and significantly increased labial root tipping from the central incisors to the canines. The maxillary central incisors showed both crown and root distal displacement, while the lateral incisors and canines showed mesial crown tipping and distal root tipping. The maxillary incisors and canines showed both crown and root occlusal extrusion (Table 3). Finally, at a lever arm length of 10 mm, the maxillary incisors and canines again showed lingual crown tipping and significantly increased labial root tipping from the central incisors to the canines. The maxillary incisors showed both crown and root distal displacement while the canines showed distal crown tipping and mesial root tipping. The maxillary incisors and canines showed both crown and root occlusal extrusion (Table 3).

In the corresponding measurements performed with the lever arm located distal to the canine with the miniscrew height at 8 mm, when the length of the lever arm was 6 mm, the maxillary incisors and canines showed lingual crown tipping and labial root tipping, less mesial crown tipping and more distal root tipping when compared to the corresponding changes at a 4.5 mm miniscrew height, and both crown and root occlusal extrusion (Table 4). At a lever arm length of 8 mm, after application of the retraction force, the maxillary incisors and canines again showed lingual crown tipping and labial root tipping, both crown and root distal displacement, and both crown and root occlusal extrusion (Table 4). Finally, at a lever arm length of 10 mm, the maxillary incisors and canines showed lingual crown tipping and labial root tipping, more crown and root distal displacement than those observed with the 8 mm long lever arm, and both crown and root occlusal extrusion (Table 4).

In another set of measurements obtained after application of the retraction force with the lever arm located between the lateral incisor and canine with a miniscrew height of 4.5 mm, when the length of the lever arm was 6 mm, the maxillary incisors showed lingual crown tipping and labial root tipping while the canines showed labial crown tipping and lingual root tipping. The maxillary incisors and canines showed mesial crown tipping and distal root tipping. The maxillary incisors also showed both crown and root occlusal extrusion, while the canines showed both crown and root intrusion (Table 5). At a lever arm length of 8 mm, the maxillary incisors again showed lingual crown tipping and labial root tipping while the canines also showed labial crown tipping and lingual root tipping. The maxillary incisors and canines showed mesial crown tipping and distal root tipping. The maxillary incisors showed both crown and root occlusal extrusion, while the canines showed both crown and root apical intrusion (Table 5). Finally, at a lever arm length of 10 mm, the maxillary incisors still showed lingual crown tipping and labial root tipping and the canines showed labial crown tipping and lingual root tipping. The maxillary incisors showed mesial crown tipping and distal root tipping, while the canines showed both crown and root distal displacement. The maxillary incisors showed both crown and root occlusal extrusion, while the canines showed both crown and root apical intrusion (Table 5).

In the next set of measurements obtained after application of a retraction force with the lever arm located between the lateral incisor and canine with the miniscrew height at 8 mm, when the length of the lever arm was 6 mm, the maxillary incisors showed lingual crown tipping and labial root tipping while the canines showed labial crown tipping and lingual root tipping. The maxillary incisors and canines showed mesial crown tipping and distal root tipping. The maxillary incisors showed both crown and root occlusal extrusion, while the canines showed both crown and root apical intrusion (Table 6). At a lever arm length of 8 mm, the maxillary incisors again showed lingual crown tipping and labial root tipping and the canines showed labial crown tipping and lingual root tipping. The maxillary incisors showed mesial crown tipping and distal root tipping, while the canines showed both crown and root distal displacement. The maxillary incisors again showed both crown and root occlusal extrusion, and the canines showed both crown and root apical intrusion (Table 6). Finally, at a lever arm length of 10 mm, the maxillary incisors again showed lingual crown tipping and labial root tipping and the canines showed labial crown tipping and lingual root tipping. Similar to the measurements with a length of 8 mm, the maxillary incisors showed mesial crown tipping and distal root tipping and the canines showed both crown and root distal displacement. The maxillary incisors showed both crown and occlusal extrusion, while the canines showed apical crown and root intrusion (Table 6).

## 4. Discussion

The results of the current study showed that all points of force application used generated lingual crown tipping with extrusion of the anterior maxillary teeth. However, the 8 mm miniscrew height with the lever arm located between the lateral incisor and canine resulted in the least amount of these patterns.

In lingual en masse retraction, the six anterior teeth are retracted as one unit because the lingual archwires have mushroom-shaped curves at the distal side of the canines [15]. Moreover, any space between the anterior teeth is not considered esthetic in lingual orthodontics [16]. With utilizing the biomechanical considerations of lingual appliances, skeletal malocclusions and craniofacial appearances can be treated and enhanced [17].

During closure of extraction spaces in lingual orthodontics, loss of torque control of the anterior teeth occurs, causing a vertical bowing effect that includes lingual tipping of the maxillary incisors [18]. Liang et al. [19] performed a 3D finite element study to compare lingual and labial orthodontics and found that under the same loading, the maxillary incisors showed lingual crown tipping in lingual orthodontics while bodily translation occurred in labial orthodontics. Furthermore, in a systemic review by Ata-Ali et al. [20], treatment with lingual appliances tended to tip incisors by generating a lingual crown torque. Feng et al. [8] conducted a 3D finite element study comparing two different lever arm locations with a single miniscrew position and concluded that lever arms located between the lateral incisor and canine can yield better anterior tooth torque control than lever arms located distal to the canine but still cannot achieve reasonable torque of the anterior maxillary teeth without additional torque control methods. In agreement with our findings, when the lever arm location was distal to the canine, the maxillary incisors and canines showed lingual crown tipping and labial root tipping, which increased as the length of the lever arm increased from 6 mm to 10 mm, with no difference between the 4.5 and 8 mm miniscrew heights.

Nevertheless, when the lever arm location was between the lateral incisor and canine, the maxillary incisors showed less lingual crown and labial root tipping than those seen with the lever arm located distal to the canine, and these tipping patterns were lesser when the miniscrew height was 8 mm than at 4.5 mm. However, the maxillary canines showed labial crown tipping and lingual root tipping, which was also lesser when the miniscrew height was 8 mm. This may be attributed to the transverse bowing effect of the lingual retraction force on the archwire [16].

Stamm et al. [21] found that 10° of torque loss caused 1.2 mm of extrusion of the maxillary incisors. In our study, the maxillary incisors extruded with all points of force application, but the extrusion tendencies were less when the lever arm was located mesial to the canines with the 8 mm miniscrew height. Hence, controlling the torque of the anterior teeth helps preventing the bite from deeping during lingual retraction.

Anchorage control is essential for successful orthodontic treatment because loss of anchorage can yield poor treatment outcomes. Miniscrews in lingual orthodontics can preserve anchorage and ultimately lead to good treatment outcomes [22]. By combining miniscrews and lingual lever arms in the retraction system, different movement patterns can be obtained with different lengths and different positions of the lever arms and miniscrews [23].

The posterior palate is considered a suitable location for miniscrew insertion [24]. Specifically, the palatal alveolus between the first and second molars offers a large interradicular space and wide cortical plate [25, 26]. However, injury to the greater palatine artery is a risk factor associated with insertion of a miniscrew in that area. Tavelli et al. [27] identified the safety zone between the cementoenamel junction of the maxillary molars and the greater palatine artery to be 13.9 ± 1 mm. In our 3D models, the miniscrews were placed at 4.5 mm and 8 mm from the archwire plane, which was approximately 4 and 6 mm, respectively, from the cementoenamel junction within the identified safety zone.

Finally, our study was limited in that it investigated initial displacements only. Although this study considered initial displacements with different retraction force directions rather than continuous, it provided some guidance for clinical treatment planning in lingual orthodontics and characterized the versatility of the 3D finite element method. Future continuous displacement finite element analysis studies and studies in a clinical setting are recommended to verify the results of this study.

## 5. Conclusions

It can be concluded that despite the limitation mentioned in this study, placing the lever arm between lateral incisor and canine with miniscrew height 8 mm could be the preferred line of action of force with additional torque control methods during en masse retraction in lingual treatment.

## Figures and Tables

**Table 1:** The numbers of node and element for each model.

	Miniscrew height: 4.5 mm	Miniscrew height: 8 mm
Lever arm: 6 mm distal to canine	Number of nodes = 132585 Number of elements = 560415	Number of nodes = 133098 Number of elements = 563514
Lever arm: 6 mm mesial to canine	Number of nodes = 132497 Number of elements = 560079	Number of nodes = 133010 Number of elements = 563178
Lever arm: 8 mm distal to canine	Number of nodes = 132776 Number of elements = 560962	Number of nodes = 133289 Number of elements = 564061
Lever arm: 8 mm mesial to canine	Number of nodes = 132749 Number of elements = 560804	Number of nodes = 133262 Number of elements = 563903
Lever arm: 10 mm distal to canine	Number of nodes = 132331 Number of elements = 559528	Number of nodes = 132844 Number of elements = 562627
Lever arm: 10 mm mesial to canine	Number of nodes = 132540 Number of elements = 560195	Number of nodes = 133053 Number of elements = 563294

**Table 2:** Material properties of the models.

	Young's modulus (MPa)	Poisson's ratio
Tooth	20000	0.30
Cortical bone	13700	0.30
PDL	0.05	0.30
Cancellous bone	1600	0.30
Titanium	110000	0.34
Stainless steel	200000	0.30
Alveolar bone	2000	0.30

**Table 3:** Initial tooth displacements following the application of retraction force (*X*: +lingual, -labial; *Y*: +mesial, -distal; *Z*: +occlusal, -apical).

	Lever arm distal to canine (*μ*m)																	
	Miniscrew height: 4.5 mm																	
	Lever arm length: 6 mm						Lever arm length: 8 mm						Lever arm length: 10 mm					
	*X*		*Y*		*Z*		*X*		*Y*		*Z*		*X*		*Y*		*Z*	
	Root	Crown	Root	Crown	Root	Crown	Root	Crown	Root	Crown	Root	Crown	Root	Crown	Root	Crown	Root	Crown
Central incisor	-3.92	74.91	-39.12	530.20	11.83	128.26	-4.41	88.37	-45.35	588.40	780.986	131.06	-4.95	106.97	-51.62	641.82	16.77	122.13
Lateral incisor	-5.16	77.72	-11.83	499.13	13.68	70.64	-5.54	95.63	-11.28	563.43	28.38	69.14	-5.92	112.17	-9.77	610.50	47.77	53.08
Canine	-1.81	375.91	-17.09	90.55	9.22	28.89	-1.25	500.69	-6.38	36.99	17.88	30.10	-4.0	639.75	-3.0	-59.21	28.20	51.27

**Table 4:** Initial tooth displacements following the application of retraction force (*X*: +lingual, -labial; *Y*: +mesial, -distal; *Z*: +occlusal, -apical).

	Lever arm distal to canine (*μ*m)																	
	Miniscrew height: 8 mm																	
	Lever arm length: 6 mm						Lever arm length: 8 mm						Lever arm length: 10 mm					
	*X*		*Y*		*Z*		*X*		*Y*		*Z*		*X*		*Y*		*Z*	
	Root	Crown	Root	Crown	Root	Crown	Root	Crown	Root	Crown	Root	Crown	Root	Crown	Root	Crown	Root	Crown
Central incisor	-3.25	73.21	-52.35	530.01	10.47	107.97	-3.96	93.43	-60.62	608.97	26.95	107.54	-4.80	116.88	-68.30	667.58	46.70	81.83
Lateral incisor	-4.68	66.51	-24.30	474.38	33.73	51.82	-5.71	83.95	-18.62	543.86	51.37	40.80	-6.10	107.68	-21.84	602.97	74.54	24.11
Canine	-5.48	446.97	-23.62	26.45	2.36	45.92	-5.06	638.91	-11.98	-83.46	9.86	88.15	-7.06	752.63	-9.44	-151.54	22.69	82.41

**Table 5:** Initial tooth displacements following the application of retraction force (*X*: +lingual, -labial; *Y*: +mesial, -distal; *Z*: +occlusal, -apical).

	Lever arm mesial to canine (*μ*m)																	
	Miniscrew height: 4.5 mm																	
	Lever arm length: 6 mm						Lever arm length: 8 mm						Lever arm length: 10 mm					
	*X*		*Y*		*Z*		*X*		*Y*		*Z*		*X*		*Y*		*Z*	
	Root	Crown	Root	Crown	Root	Crown	Root	Crown	Root	Crown	Root	Crown	Root	Crown	Root	Crown	Root	Crown
Central incisor	-2.66	21.19	-12.20	74.07	13.77	3.88	-2.97	23.42	-9.35	-26.77	71.95	90.17	-3.12	20.53	-5.11	-189.63	80.13	123.65
Lateral incisor	-5.71	44.54	-8.23	104.58	17.36	7.0	-6.68	46.38	-7.14	5.55	65.36	80.25	-7.99	43.20	-4.96	-146.69	70.42	101.86
Canine	10.84	-116.20	-8.65	124.93	-36.70	-5.16	9.45	-149.42	-3.73	26.89	-53.41	-65.62	8.77	-200.47	1.76	-106.26	-51.29	-44.27

**Table 6:** Initial tooth displacements following the application of retraction force (*X*: +lingual, -labial; *Y*: +mesial, -distal; *Z*: +occlusal, -apical).

	Lever arm mesial to canine (*μ*m)																	
	Miniscrew height: 8 mm																	
	Lever arm length: 6 mm						Lever arm length: 8 mm						Lever arm length: 10 mm					
	*X*		*Y*		*Z*		*X*		*Y*		*Z*		*X*		*Y*		*Z*	
	Root	Crown	Root	Crown	Root	Crown	Root	Crown	Root	Crown	Root	Crown	Root	Crown	Root	Crown	Root	Crown
Central incisor	-2.21	24.18	-21.63	25.17	41.31	32.87	-2.59	28.07	-18.18	-93.80	40.04	45.10	-2.90	24.20	-13.05	-262.33	111.17	167.42
Lateral incisor	-5.82	34.12	-18.54	26.93	41.86	32.96	-7.02	38.75	-16.27	-87.08	38.33	35.31	-8.75	35.45	-12.42	-238.36	95.53	14.34
Canine	6.71	-53.56	-11.14	21.52	-47.69	-41.91	6.11	-102.46	-6.79	-125.78	-41.96	-21.08	5.79	-160.09	-1.84	-255.45	-60.20	-77.20

## Data Availability

The research article data used to support the findings of this study are included within the article.
